# Impact of Exposure to Disinfectants on Presence of Efflux Pump Genes and Antibiotic Resistance Profiles in *Escherichia coli* Isolates

**DOI:** 10.3390/microorganisms13122700

**Published:** 2025-11-26

**Authors:** Fernanda Borges Barbosa, Beatriz Rodrigues Takeda, Gabriella Garcia Ilion Vicentini, Gabriel Gandolfi, Victória Galdino Pavlenco Rocha, Leticia Soares Franco, Marcos Paulo Vieira Cunha, Terezinha Knöbl

**Affiliations:** School of Veterinary Medicine and Animal Science, University of São Paulo, São Paulo 05508-270, Brazil; fernanda.borges.barbosa@usp.br (F.B.B.);

**Keywords:** *qacE*, *qacH*, *ydgF*, QAC, poultry

## Abstract

Disinfectant tolerance in bacteria may be related to exposure to subinhibitory concentrations of disinfectants, which may activate efflux pumps capable of expelling antimicrobial compounds. The aim of this study was to evaluate the impact of disinfection on the presence of efflux pump genes and the resistance profile of *Escherichia coli* from commercial laying farms employing different disinfection protocols. The *emrE*, *qacE*, *qacEΔ1*, *qacH*, *sugE(c)*, *ydgE*, *ydgF*, and class 1 integron (*intl1*) genes were investigated using PCR. Susceptibility to 17 antibiotics was assessed, including β-lactams, fluoroquinolones, aminoglycosides, and tetracyclines. Disinfectant exposure was significantly associated with higher frequencies of *qacE* and *qacH*, and a reduced frequency of *ydgF*. Moreover, resistance to ampicillin, trimethoprim–sulfamethoxazole, and doxycycline was significantly more frequent in *E. coli* isolated from chickens exposed to disinfectants. These findings indicate that disinfectant use can select for *E. coli* carrying efflux pump genes and resistance genes, favoring the survival and dissemination of tolerant and resistant strains in poultry production. Continuous monitoring and the development of disinfection strategies that minimize selective pressures are crucial for limiting the spread of antimicrobial resistance at the animal–human–environment interface.

## 1. Introduction

Animal production faces several challenges, including the emergence of health problems due to high confinement rates [1]. To reduce microbial loads, biosecurity protocols must be implemented, including cleaning and disinfection measures, as well as guidelines for performing a sanitary void. Disinfectants such as quaternary ammonium compounds (QACs) and glutaraldehyde are widely used in poultry farms because of their effectiveness against major poultry pathogens such as *Salmonella* spp. Avian Pathogenic *E. coli* (APEC), *Pasteurella multocida*, *Avibacterium paragallinarum*, Newcastle disease, and avian influenza virus [2].

QACs are powerful cationic disinfectants widely used for cleaning and disinfecting hospitals, clinics, schools, industries, restaurants, and veterinary facilities. QACs are effective disinfectants, but their capacity to kill mycobacteria, non-enveloped viruses, and bacterial spores is limited and depends on formulation, concentration, use conditions, and exposure time. Their mechanisms of action include inactivation of energy-producing enzymes, protein denaturation, and cell membrane breakdown [3]. These products are commonly used at concentrations between 1000 and 5000 ppm alongside detergent formulations, and they must be thoroughly rinsed off after use [4].

Glutaraldehyde is an aldehyde broad-spectrum disinfectant with sporicidal, virucidal, fungicidal, and bactericidal activities, but with reduced efficacy against mycobacteria. Glutaraldehyde can disrupt bacterial biofilms, which are common on farm surfaces and equipment. It targets the proteins and enzymes of the bacterial cell, affecting metabolism and leading to death of the microorganism. It is used at concentrations between 1 and 2% [5].

QACs and glutaraldehyde are relatively inexpensive, easy to apply, and widely available. Their combination can provide a synergistic effect, improving the overall disinfection efficacy, and has been extensively used in poultry biosecurity programs for disinfection of facilities, equipment, and vehicles. However, the drawbacks and risks associated with their intensive use include toxicity, occupational hazards, environmental persistence, and the selection of bacteria with increased tolerance to disinfectants and cross-resistance to antibiotics [6].

Antibiotics have traditionally been used to treat infections and promote growth [2]. However, since 2018, the ban on the use of certain antibiotics as growth promoters has been enforced in Brazil [7]. In response to this restriction, the use of disinfectants has significantly increased to prevent microbial proliferation, with daily applications occurring on farms through spraying [8].

The misuse of antimicrobials can lead to resistance, defined as the ability of microorganisms to resist the effects of antimicrobial agents [9]. Antimicrobial resistance is a natural phenomenon arising from mutations or horizontal gene transfer [10]. Antimicrobial resistance genes can be carried by chromosomes, plasmids, or transposons [11].

Disinfectant tolerance may be related to inappropriate use, such as exposing microorganisms to subinhibitory concentrations. This exposure may result in the development of mutated enzymes or changes in the cell membrane, metabolic cascades, or gene expression patterns related to biofilm formation or efflux pumps [12].

Efflux pumps are an important mechanism of resistance to QACs. Using an energy- or proton-dependent mechanism, the pumps can reduce the concentration of antimicrobial compounds inside the bacterial cell, transferring the compounds to the external environment. This mechanism can be influenced by the external concentration of QACs [12].

Resistance to QACs may be related to the presence of genes such as members of the small multidrug resistance (SMR) family or the major facilitator superfamily (MFS). The *qacG*, *qacH* and *qacJ* genes from the SMR family are present on plasmids smaller than 3 kb, and the *qacA* and *qacB* genes from the MFS are present on large plasmids [2]. The *sugE(p)* gene (also named *gdx*) is a member of the SMR family and is also carried by plasmids. Some genes related to resistance are present in the chromosome, such as the genes *emrE*, *sugE(c)* (also named as *gdx*), *ydgE*, and *ydgF*, which are members of the SMR family. The frequency of reports involving these genes has been increasing [13].

Resistance to QACs is also associated with antimicrobial resistance due to the presence of genes conferring resistance to QACs and antibiotics on the same genetic elements. [6,14]. Frequently, *qac* genes, which contribute to QAC resistance, are found in class 1 integrons, which also carry gene cassettes containing antibiotic resistance genes [12].

The aim of this study was to evaluate the impact of disinfection on the presence of efflux pump genes and the antibiotic resistance profile of *Escherichia coli* isolated from chickens in commercial laying farms subjected to different disinfection protocols in comparison with *E. coli* from chickens in free-range production. The three disinfectant protocols were applied at least three times a week and were compared to a non-exposure to disinfectant group.

## 2. Materials and Methods

### 2.1. Ethical Approval

This study was approved by the Ethics Committee on the Use of Animals (CEUAX—protocol 3328060320) of the School of Veterinary Medicine and Animal Science of the University of São Paulo (FMVZ-USP).

### 2.2. Study Design and Sampling

This study was designed as an experimental field study carried out under commercial farm conditions. Sampling followed a convenience design and was based on the availability of laying farms in the state of São Paulo, Brazil, between 2019 and 2022. Farms were selected according to their disinfection practices, allowing for a comparison of sites applying distinct protocols and one site without any disinfection (non-exposed group).

Cloacal swabs were collected from individual chickens (*Gallus gallus*) housed on farms that were disinfected by spraying a quaternary ammonium compound (benzalkonium chloride) combined with glutaraldehyde and chickens present in farms without disinfection (non-exposed group). The samples were transported to the laboratory under refrigerated conditions (4–8 °C) and processed within 24 h of collection to ensure bacterial viability.

A total of 123 cloacal swabs were collected across four groups, classified according to the disinfection protocol. Protocol 1 involved disinfection three times a week, protocol 2 involved disinfection every other day, and protocol 3 involved disinfection twice a day. The reference group consisted of free-range production; in contrast, protocols 1 to 3 consisted of Californian-type cages. The study groups are described in Table 1.

### 2.3. Bacterial Isolation and Identification

The cloacal swabs were incubated in peptone water at 37 °C for 24 h and subsequently streaked onto MacConkey agar (DB^®^, Detroit, MI, USA), followed by incubation for an additional 24 h at 37 °C. From each plate, a single circular, flat, lactose-fermenting colony (indicative of *Escherichia coli*) was selected to avoid duplicate sampling. Isolates were identified using matrix-assisted laser desorption/ionization time-of-flight mass spectrometry (MALDI-TOF MS). Protein extraction was performed using the formic acid–acetonitrile protocol. The mass spectra of the isolates were compared with reference spectra using the Biotyper™ software (MALDI Biotyper CA Systems 3.0, Bruker Daltonik, Bremen, Germany). Identification scores were interpreted according to the manufacturer’s guidelines: values ≥ 2.0 indicated identification at both the genus and species levels, whereas scores between 1.7 and 2.0 indicated genus-level identification [15].

### 2.4. DNA Extraction and PCR Assays

All strains were subjected to conventional Polymerase Chain Reaction (PCR) to detect disinfectant resistance genes. DNA extraction was performed by boiling. PCRs were prepared using 11.8 µL of ultrapure water, 0.3 µM of each primer (forward and reverse), 4 µL of FIREPol^®^ Master Mix (Solis BioDyne^®^, Tartu, Estonia), and 3 µL of the DNA template. The FIREPol^®^ master mix is composed of buffer (0.4 M Tris-HCl, 0.1 M (NH_4_)_2_SO_4_, 0.1% (*w*/*v*) Tween 20), magnesium (12.5 mM MgCl_2_), and phosphate deoxyribonucleotides (dNTPs) (1 µM). The primers used are described in Table 2. Amplification was carried out under the following conditions: initial denaturation at 95 °C for 5 min; 30 cycles of 95 °C for 30 s, annealing at the specific temperature for each primer (Table 2) for 30 s, and 72 °C for 1 min; followed by a final extension at 72 °C for 7 min. The PCR products were separated on a 1.5% agarose gel, stained with BlueGreen^®^ (Nova Biotecnologia, São Paulo, Brazil), and visualized under UV illumination. Previously sequenced strains were used as negative and positive controls.

### 2.5. Antibiotic Susceptibility Testing

All strains were subjected to phenotypic evaluations of antibiotic susceptibility using the Kirby–Bauer disk diffusion method on Mueller–Hinton agar, following the Clinical and Laboratory Standards Institute guidelines [20]. The tested agents and concentrations were as follows: amoxicillin–clavulanic acid (20:10 µg), ampicillin (10 µg), aztreonam (30 µg), cefotaxime (30 µg), cefoxitin (30 µg), ceftiofur (30 µg), cefazolin (30 µg), ciprofloxacin (5 µg), chloramphenicol (30 µg), trimethoprim–sulfamethoxazole (25 µg), doxycycline (30 µg), fosfomycin (200 µg), gentamicin (10 µg), meropenem (10 µg), piperacillin–tazobactam (100:10 µg), and tigecycline (15 µg). The American Type Culture Collection (ATCC) *Escherichia coli* 8462234 strain was used as the quality control.

Multidrug-resistant (MDR) *E. coli* strains were defined as strains resistant to one or more antibiotics from three or more classes of antimicrobials. Extensively resistant (XDR) strains were defined as strains resistant to one or more antibiotics in all but two or fewer categories, as proposed by Magiorakos et al. (2011). Pan drug-resistant (PDR) strains were defined as strains resistant to all the agents listed by Magiorakos et al. (2011) [21].

### 2.6. Statistical Analysis

Statistical analysis was performed in RStudio (v4.4.0) using Fisher’s Exact Test on 2 × 2 contingency tables to compare resistance frequencies between the non-exposed and disinfection protocol groups, considering a significance level of 5% (*p*-value < 0.05). Comparisons were first conducted using the weighted means of protocols 1–3 and individual protocols (Non-exposed × Protocol).

## 3. Results

A total of 123 *E. coli* strains were analyzed, considering one colony per sampled bird (Table 1).

### 3.1. Detection of Disinfectant Resistance Genes

The frequencies of efflux pump genes in the different groups are summarized in Table 3. Compared with the non-exposed group, the frequencies of the *ydgE*, *ydgF*, *qacEΔ1*, and *intI1* genes were lower in the disinfectant-exposed groups, with statistically significant differences for the *ydgF* gene. When the Non-exposed group was compared to the weighted mean of protocols 1–3, higher frequencies of the *emrE*, *qacE*, *qacH* and *sugE(c)* genes were observed, with statistically significant differences between the frequencies of the *qacE* and *qacH* genes. Therefore, there was a relationship between the frequencies of the *qacE*, *qacH*, and *ydgF* genes and the disinfection protocol applied.

In the Non-exposed group (n = 28), the most frequently detected genes were *ydgF* (96.43%) and *ydgE* (92.86%), both chromosomally encoded members of the small multidrug resistance (SMR) family. The integrase gene *intl1* was also common, detected in 85.71% of isolates, while *sugE(c)* was found in 53.57%. The plasmid-borne genes *qacEΔ1* and *qacE* were identified in 25.0% and 10.71% of isolates, respectively. Only one isolate (3.57%) carried *emrE*, and no isolates carried *qacH*.

Among the disinfectant-exposed groups, a shift in gene prevalence was observed. In protocol 1 (n = 20), the most frequent genes were *intl1* (n = 19, 95%) and *ydgE* (n = 17, 85%). Other less frequent genes were *sugE*(c) (n = 6, 30%), *emrE* (n = 3, 15.00%), *qacEΔ1* (n = 2, 10%), and *qacH* (n = 1, 5%). None of the strains were positive for the *qacE* and *ydgF* genes.

In protocol 2 (n = 53), the isolates exhibited a more diverse profile. The *ydgE* gene remained the most frequent (n = 49, 92.45%), followed by *sugE*(c) (n = 33, 73.58%), *intl1* (n = 34, 64.15%), *qacE* (n = 30, 56.6%), and *ydgF* (n = 23, 43.40%). *qacH was present in* 22.64% (n = 12) while *emrE* and *qacEΔ1* were present in 16.98% (n = 9).

**Figure 1:**
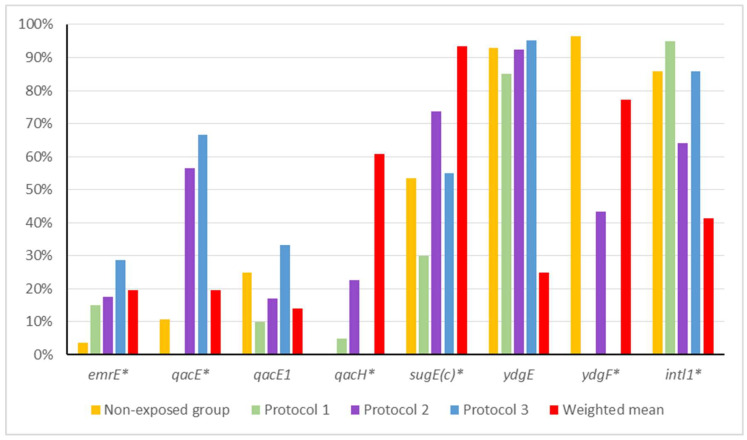
Frequencies of efflux pump and integron genes in Non-exposed and disinfectant-exposed groups. * Genes showing statistically significant differences between non-exposed group and protocols 1 to 3.

In protocol 3 (n = 22), the frequencies of the resistance genes, in decreasing order, were *ydgE* (n = 20, 90.91%), *intl1* (n = 18, 81.82%), *sugE(c)* (n = 11, 50%), *qacE* (n = 8, 36.36%), *qacEΔ1* (n = 7, 31.82%), and *emrE* (n = 6, 27.27%). No strain was positive for the *qacH* and *ydgF* genes. When data from all disinfectant-exposed groups were combined into a weighted mean, significant differences emerged compared with the Non-exposed group. The genes *emrE*, *qacE*, and *qacH* were significantly more frequent in the exposed groups, while *ydgF* was significantly less frequent (*p* < 0.0001). This suggests that disinfection pressures selectively enriched the presence of certain efflux pump genes while reducing the prevalence of others (Figure 1).

Pairwise comparisons revealed specific associations for each protocol. In protocol 1, isolates carried the *ydgF* gene at a significantly lower frequency than the Non-exposed group (*p* < 0.0001). In protocol 2, differences were detected for *qacE* (*p* = 0.00004), *qacH* (*p* = 0.0063), and *ydgF* (*p* < 0.0001). In protocol 3, there were significant differences between the isolates and the Non-exposed group regarding the presence of the *emrE* gene (*p* = 0.034), as well as the *qacE* (*p* < 0.0001) and *ydgF* (*p* < 0.0001) genes.

### 3.2. Antibiotic Resistance Phenotypes

We observed a higher frequency of resistance in the disinfectant-exposed groups than in the Non-exposed group for all the antibiotics tested (Table 4), with statistically significant differences for ampicillin, trimethoprim–sulfamethoxazole, and doxycycline.

In the Non-exposed group, resistance was rare, with only 7.14% (n = 2) showing resistance to doxycycline. By contrast, isolates from protocol 1 exhibited high levels of resistance. Resistance to doxycycline was the most frequent (n = 13, 65.0%), followed by ampicillin (n = 6, 30.0%), trimethoprim–sulfamethoxazole (n = 5, 25.0%), amoxicillin–clavulanic acid (n = 2, 10.00%), chloramphenicol (n = 2, 10.0%), aztreonam (n = 1, 5.0%), cefazolin (n = 1, 5.0%), and ciprofloxacin (n = 1, 5.0%). All strains were susceptible to cefoxitin, ceftiofur, gentamycin, piperacillin–tazobactam, and tigecycline.

In protocol 2, a broader resistance profile was observed, though the resistance levels were slightly lower than in protocol 1. The most common resistances were against trimethoprim–sulfamethoxazole (n = 19, 35.85%), ampicillin (n = 16, 30.19%), doxycycline (n = 16, 30.19%), cefazolin (n = 6, 11.32%), chloramphenicol (n = 6, 11.32%), piperacillin–tazobactam (n = 3, 5.66%), amoxicillin–clavulanic acid (n = 3, 5.66%), tigecycline (n = 2, 3.77%), aztreonam (n = 1, 1.89%), and cefoxitin (n = 1, 1.89%). All strains were susceptible to ceftiofur, ciprofloxacin, and gentamycin.

In protocol 3, resistance was less frequent but still broader than in the Non-exposed group. The highest resistance rate was to doxycycline (n = 6, 27.27%), aztreonam (n = 4, 18.18%), cefazolin (n = 3, 13.64%), tigecycline (n = 2, 9.09%), ceftiofur (n = 1, 4.55%), trimethoprim–sulfamethoxazole (n = 1, 4.55%), and gentamicin (n = 1, 4.55%). All strains were susceptible to amoxicillin–clavulanic acid, ampicillin, cefoxitin, ciprofloxacin, chloramphenicol, and piperacillin–tazobactam.

**Figure 2:**
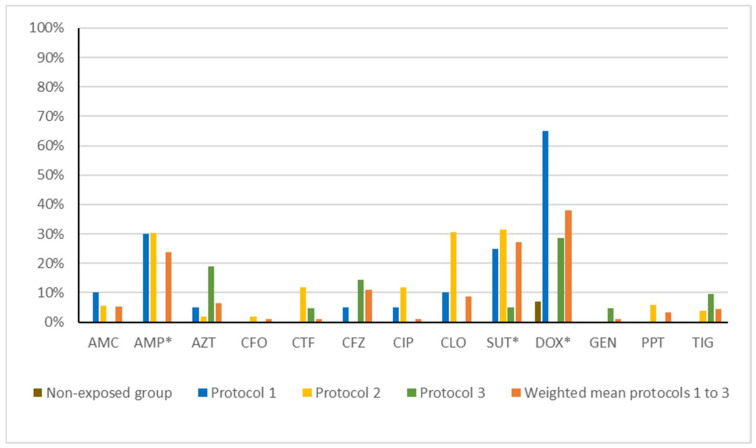
Frequency of antibiotic resistance in Non-exposed and disinfectant-exposed groups. Note: AMC: amoxicillin–clavulanic acid (30 µg); AMP: ampicillin (10 µg); AZT: aztreonam (30 µg); CFO: cefoxitin (30 µg); CTF: ceftiofur (30 µg); CFZ: cefazolin (30 µg); CIP: ciprofloxacin (5 µg); CLO: chloramphenicol (30 µg); SUT: trimethoprim–sulfamethoxazole (25 µg); DOX: doxycycline (30 µg); GEN: gentamicin (10 µg); PPT: piperacillin–tazobactam (110 µg); and TIG: tigecycline (15 µg). No resistance to cefotaxime (30 µg), fosfomycin (200 µg), or meropenem (10 µg) was observed. * Antibiotics showing statistically significant differences between Non-exposed group and protocols 1 to 3.

When the disinfectant-exposed groups were combined, the resistance rates were significantly higher than in the Non-exposed group for several antibiotics. In particular, resistance to ampicillin (23.2%, *p* = 0.0035), trimethoprim–sulfamethoxazole (26.3%, *p* = 0.0009), and doxycycline (36.8%, *p* = 0.002) showed strong statistical associations with disinfectant exposure (Figure 2). No strain in the Non-exposed group were MDR, compared with 10.0% (n = 2), 18.9% (n = 10), and 4.6% (n = 1) in protocols 1, 2, and 3, respectively. We observed statistically significant differences in MDR prevalence between the Non-exposed group and protocol 2 (*p* = 0.0128).

Pairwise analyses revealed specific associations for each protocol. In protocol 1, the resistance rates were significantly higher for ampicillin (*p* = 0.0031), trimethoprim–sulfamethoxazole (*p* = 0.009), and doxycycline (*p* < 0.0001) compared with the Non-exposed group. Similarly, in protocol 2, isolates showed significantly increased resistance to ampicillin (*p* = 0.0007), trimethoprim–sulfamethoxazole (*p* < 0.0001), and doxycycline (*p* = 0.0234). In protocol 3, significant differences were observed for aztreonam (*p* = 0.0317).

## 4. Discussion

Previous studies, such as Zou et al. (2014), have demonstrated that the use of QACs in food-processing environments can select for resistant bacterial strains carrying disinfectant resistance genes [18]. In agreement with these findings, our study identified a significant association between disinfectant exposure protocols and the frequency of genes encoding efflux pumps. Specifically, isolates from farms subjected to intensive disinfection protocols exhibited a higher prevalence of *emrE*, *qacE*, *qacH*, and *sugE(c)* compared to isolates from farms without routine disinfection. Notably, *emrE* and *sugE(c)* are chromosomally encoded SMR-family efflux pumps, whereas *qacE* and *qacH* are located on mobile genetic elements such as plasmids and integrons. These genes confer resistance to a variety of QACs and related compounds, including cetyltrimethylammonium bromide, hexadecyltrimethylammonium bromide, cetylpyridinium chloride, and benzalkonium chloride. The elevated frequency of these genes in disinfectant-exposed groups indicates that repeated chemical disinfection can act as a strong selective pressure, favoring the maintenance and dissemination of specific resistance determinants, particularly those carried on mobile genetic elements [18].

In a related study, Jiang et al. (2017) reported a higher frequency of *sugE(c)*, *ybgE/ybgF*, *emrE*, and *qacEΔ1* than observed in our study [16]. They reported similar frequencies for *qacE* and *qacH* but observed lower prevalence of *intl1*. Their study, which surveyed retail meats, highlighted the diversity of resistance gene profiles across different meat-production systems [16]. In our study, we observed higher frequencies of the *emrE*, *qacE*, *qacH* and *sugE(c)* genes, suggesting selection for specific SMR-family pumps under higher disinfection pressures. In addition, the lower frequency of the *ydgF* gene suggests that high disinfection pressures may discourage the expression of SMR pumps. These efflux pumps offer a fast solution to stress, acting as an effective mechanism to resist the effects of drugs [22].

The differential prevalence of plasmid-mediated versus chromosomally encoded resistance genes underscores the complex and context-dependent nature of bacterial adaptation to disinfection protocols. Repeated exposure to subinhibitory concentrations of QACs has been shown to drive genetic changes, including the overexpression of efflux pumps, increased biofilm formation, and changes to metabolic pathways [23]. This indicates that disinfectant pressure can simultaneously enrich mobile resistance determinants while diminishing the relative importance of chromosomal resistance mechanisms.

Of particular concern is the high prevalence of *sugE(c)* across all studied groups. *sugE*, which is related to guanidinium-selective small multidrug resistance efflux pumps, confers high tolerance to QAC disinfection. Even though there was no statistically significant difference in the presence of *sugE(c)*, the high frequency in all studied groups represents a concern. Slipski et al. (2020) found a high prevalence of *sugE*, which was related to the high disinfection tolerance of biofilms, in environments containing small guanidinium. The authors found that strains harboring *sugE(c)* and present in biofilms required higher QAC concentrations for eradication, highlighting the role of biofilms in disinfectant resistance [23].

Despite several studies showing that QAC resistance is not related to cross-resistance to antimicrobials, Zou et al. (2014) found an association between the presence of *qacEΔ1* and *sugE(p)* and sulphonamide resistance, which is coded by *sul1* [18]. Consistent with this observation, our study found that 38.88% (n = 7/18) of the strains positive for *qacEΔ1* displayed resistance to trimethoprim–sulfamethoxazole and none of the Non-exposed group strains displayed resistance to trimethoprim–sulfamethoxazole, reinforcing this association. We also found increased antibiotic resistance frequencies in the disinfectant-exposed groups, notably for ampicillin, trimethoprim–sulfamethoxazole, and doxycycline, with statistically significant differences compared to the non-exposed group. Overall, these findings demonstrate that disinfectant exposure was consistently associated with increased antimicrobial resistance, particularly to β-lactams (ampicillin and aztreonam), tetracyclines (doxycycline), and sulfonamides (trimethoprim–sulfamethoxazole). However, this observed selection may also be attributable to antibiotic usage practices, considering that antibiotic administration data for the studied farms was unavailable. Without this information, it is not possible to definitively attribute the observed resistance patterns to disinfectant pressure alone, and the possibility of co-selection driven by antibiotic use cannot be excluded.

A limitation of the study was the absence of data on antibiotic usage, which could have contributed to the higher resistance rates observed in the disinfectant-exposed groups. Nevertheless, these findings emphasize the need to consider the impact of disinfection protocols on resistance gene dynamics and the potential for cross-resistance or co-selection, which may compromise both disinfection efficacy and antibiotic therapy [18,22].

Besides the relationship between disinfection exposure and efflux pump genes selection, our study highlights the need for effective biosecurity strategies to control microbial contamination and limit the emergence of resistant bacterial strains in poultry production. Intensive or improper use of these disinfectants can inadvertently select for bacterial populations carrying efflux pump genes, such as *qacE*, *qacH*, *emrE*, and *sugE(c)*, which confer resistance to both disinfectants and, indirectly, certain antibiotics [18,22].

Optimizing disinfectant use requires a collective approach. First, farms should establish protocols that define the appropriate concentrations, frequency, and methods of application to minimize subinhibitory exposure that can drive resistance selection [23]. Rotating disinfectants with different mechanisms of action may also help reduce selective pressure on specific efflux pump genes, limiting the risk of development of tolerance or cross-resistance. Mechanical cleaning and biofilm disruption should be integrated with chemical disinfection, as biofilms can protect bacteria from even high concentrations of disinfectants and contribute to the persistence of resistant strains [23]. In addition, staff training is crucial to ensure that disinfectants are applied correctly and consistently, reducing the likelihood of sub-dosing or incomplete coverage that can promote resistance.

The development and implementation of guidelines for disinfectant usage in animal production are equally important. Currently, most regulatory frameworks focus primarily on antibiotic stewardship, with limited guidance on chemical disinfectants. Standardized protocols for disinfectant selection, application, and monitoring would help ensure that these compounds are used effectively without inadvertently promoting resistance. Such guidelines should also incorporate monitoring of disinfectant resistance genes alongside traditional antibiotic resistance surveillance to provide a comprehensive understanding of microbial adaptation on farms. Establishing clear thresholds for safe and effective disinfectant use would enable producers to balance the need for pathogen control with the goal of minimizing selection for resistant bacteria.

## 5. Conclusions

The data from this study demonstrate that the more intensive use of disinfectants can lead to an increase in the frequency of efflux pumps encoded by the *qacE*, *qacH*, and *ydgF* genes. The higher frequencies of these genes in groups exposed to disinfectants suggest that the selective pressure exerted by these chemical agents may favor the survival and dissemination of resistant and tolerant bacteria. These findings highlight the importance of careful management of disinfection protocols in poultry production to avoid the inadvertent promotion of multidrug-resistant strains, thus preserving the efficacy of both disinfectants and antibiotics. Future research should include data on antibiotic use to better understand the interactions between disinfection and bacterial resistance.

## Figures and Tables

**Table 1:** Study groups, production types, and disinfection practices.

Group	Production Type	Sample Year	Disinfection Protocol	Disinfection Dilution	Frequency of Disinfection	№ of Samples
Non-exposed	Free-range	2022	Without disinfection	-	-	28
Protocol 1	Caged	2021	QAC, glutaraldehyde, ethanolic aldehyde, and chemical enhancers	1:400 L	Three times a week	20
Protocol 2	Caged	2021	QAC and glutaraldehyde	1:100 L	Every other day	53
Protocol 3	Caged	2021	QAC, glutaraldehyde, ethanolic aldehyde, and chemical enhancers	1:100 L	Twice a day	22
Total						123

**Table 2:** Sequence, description, annealing temperature, amplicon size, and reference of primers used in this study.

Gene	Sequence (5′–3′)	Description	Annealing (°C)	Amplicon (pb)	Reference
*emrE*	CCTGTTATGGGCGGTAGAC TTCGTGCTCACCTTTCCTT	Efflux pumps (multidrug)	54	310	[16]
*qacE*	AGCCCCATACCTACAAAG AGCTTGCCCCTTCCGC	Efflux pumps (QAC)	55	194	[17]
*qacEΔ1*	AAGTAATCGCAACATCCG ATAAGCAACACCGACAGG	Efflux pumps (QAC)	49	140	[16]
*qacH*	TTTGGTGAGGTCGTCGCA GCCAGCCCAAACAGCATA	Efflux pumps (QAC)	54	162	[16]
*sugE(c)*	CTGCTGGAAGTGGTATGGG GCATCGGGTTAGCGGACT	Efflux pumps (QAC)—chromosomal	55	226	[18]
*ydgE*	GGCAATCGTGCTGGAAAT GGCGGCAATACCAAACCC	Efflux pumps (multidrug)	54	184	[16]
*ydgF*	ATTACCTTGTTTAGCGTTTT GGTTCACCTCCAGTTCAG	Efflux pumps (multidrug)	49	139	[16]
*intl1*	ACGAGCGCAAGGTTTCGGT GAAAGGTCTGGTCATACATG	Class 1 integron	54	150	[19]

**Table 3:** Frequencies of disinfectant resistance genes according to study group.

Group	*emrE*	*qacE*	*qacEΔ1*	*qacH*	*sugE(c)*	*ydgE*	*ydgF*	*intl1*
Non-exposed	3.57%	10.71%	25.00%	0%	53.57%	92.86%	96.43%	85.71%
Protocol 1	15.00%	0%	10.00%	5.00%	30.00%	85.00%	0%	95.00%
Protocol 2	16.98%	56.60%	16.98%	22.64%	73.58%	92.45%	43.40%	64.15%
Protocol 3	27.27% *	36.36%	31.82%	0%	50.00%	90.91%	0%	81.82%
Weighted mean (protocols 1 to 3)	18.95%	40%	18.95%	13.68%	58.95%	90.53%	24.21%	74.74%
*p*-value (Non-exposed vs. weighted mean)	0.0708	0.0031 *	0.5932	0.0385 *	0.6664	1	<0.0001 *	0.3073

* Statistically significant differences (Non-exposed × Protocol).

**Table 4:** Frequency of antibiotic resistance in Non-exposed and disinfectant-exposed groups.

Group	AMC	AMP	AZT	CFO	CTF	CFZ	CIP	CLO	SUT	DOX	GEN	PPT	TIG
Non-exposed	0%	0%	0%	0%	0%	0%	0%	0%	0%	7.14%	0%	0%	0%
Protocol 1	10.0%	30.00%	5.00%	0%	0%	5.00%	5.00%	10.00%	25.00%	65.00%	0%	0%	0%
Protocol 2	5.66%	30.19%	1.89%	1.89%	0%	11.32%	0%	11.32%	35.85%	30.19%	0%	5.66%	3.77%
Protocol 3	0%	0%	18.18%	0%	4.55%	13.64%	0%	0%	4.55%	27.27%	4.55%	0%	9.09%
Weighted mean (protocols 1 to 3)	5.26%	23.16%	6.32%	1.05%	1.05%	10.53%	1.05%	8.42%	26.32%	36.84%	1.05%	3.16%	4.21%
*p*-value (Non-exposed vs. weighted mean)	0.5875	0.0035 *	0.335	1	1	0.1145	1	0.1963	0.0009 *	0.002 *	1	1	0.5731

* Statistically significant differences. AMC: amoxicillin–clavulanic acid (30; AMP: ampicillin (10 µg); AZT: aztreonam (30 µg); CFO: cefoxitin (30 µg); CTF: ceftiofur (30 µg); CFZ: cefazolin (30 µg); CIP: ciprofloxacin (5 µg); CLO: chloramphenicol (30 µg); SUT: trimethoprim–sulfamethoxazole (25 µg); DOX: doxycycline (30 µg); GEN: gentamicin (10 µg); PPT: piperacillin–tazobactam (110 µg); and TIG: tigecycline (15 µg). No resistance to cefotaxime (30 µg), fosfomycin (200 µg), or meropenem (10 µg) was observed.

## Data Availability

The original contributions presented in this study are included in the article. Further inquiries can be directed to the corresponding author.
